# Supplementary material for: Splice-Junction-Based Mapping of Alternative Isoforms in the Human Proteome
Source: Cell Rep. Author manuscript; Available in PMC 2020 Jan 15. (PMC6961840; doi:10.1016/j.celrep.2019.11.026)

A

sp|P27816|MAP4_HUMAN|ENSG00000047849|SE2|16816|chr3|47917174|47918841|−2|r6|T1
M[15.99]YHDDDLADLVFPSSATADTSIFAGQNDPLKDSYVPLELAK q value: 0.00086699 Tr_novel:TRUE RefSeq_Nov
Search result spec prec mz: 1122.546 Actual spec prec mz: 1122.546
Fragments matched per AA: 0.488 Proportion of top 20 peaks matched: 0.15

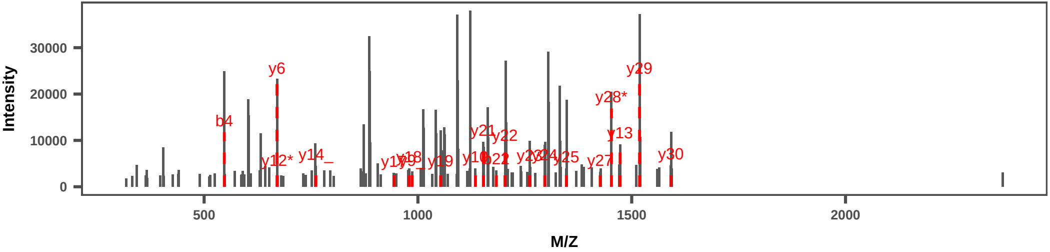

B

Scatterplot of predicted elution time
Fitting R2: 0.81
Novel peptide residual Z score: −0.0515
Number of peptides: 667

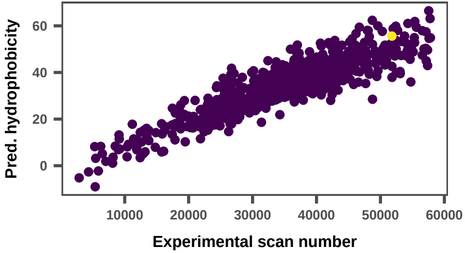

C

Distributions of residuals from best−fit line
of predicted RT vs Expt. scan number
Line: Z score of novel peptide
Z: −0.0515

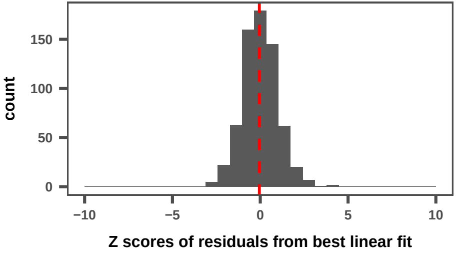

Supplement: 2 [file NIHMS1546469-supplement-2.zip › DF1/PXD010154/Ovary/Ovary_12_MAP4_MYHDDDLADLVFPSSATADTSIFAGQNDPLKDSYVPLELAK.pdf]
